# Bio-signal induced emotion monitoring and detection of anxiety: A sensor-driven approach with regression based random forest

**DOI:** 10.1016/j.mex.2025.103713

**Published:** 2025-11-10

**Authors:** Adwitiya Mukhopadhyay, Divyashree D P, Ramya C A, Hijaz Ahmad, Taha Radwan, Soumik Das

**Affiliations:** a Amrita School of Computing, Amrita Vishwa Vidyapeetham, Mysuru campus, Karnataka, India; b Sustainability Competence Centre, Széchenyi István University, Egyetem tér 1, H-9026, Győr, Hungary; c Operational Research Center in Healthcare, Near East University, Nicosia/TRNC 10, 99138 Mersin, Turkey; d Department of Mathematics, College of Science, Korea University, 145 Anam-ro, Seongbuk-gu, Seoul 02841, South Korea; e Engineered Biomaterials Research Center, Khazar University, Baku, Azerbaijan; f Department of Management Information Systems, College of Business and Economics, Qassim University, Buraydah 51452, Saudi Arabia; g School of Physical Sciences, Amrita Vishwa Vidyapeetham, Mysuru campus, Karnataka, India

**Keywords:** Regression based random forest (RBRF), Heart rate (HR), Galvanic skin response (GSR), Decision tree (DT), Machine learning (ML)

## Abstract

The present study addresses the rising importance of mental health by devel oping a novel healthcare plan. We integrate physiological data from sensors, such as Heart Rate (HR) and Galvanic Skin Response (GSR), to predict and manage anxiety. These sensors provide non-invasive insights into the com plex relationship between physiological reactions and mental well-being. To analyze the collected data, we developed a novel algorithm, Regression Based Random Forest (RBRF). Using a large-scale dataset, we empirically validated the effectiveness of our approach, achieving an impressive 95 % accuracy in identifying anxiety. Our findings demonstrate the potential of sensor-based technologies and advanced algorithms to empower individuals to proactively monitor and manage their mental health. This approach holds significant promise for improving the precision and effectiveness of mental health care.

• The study aims to improve mental healthcare by incorporating physiological data (Heart Rate and Galvanic Skin Response) to detect and potentially treat anxiety.
• Employs a novel algorithm, Regression Based Random Forest (RBRF), to analyze the collected data and identify anxiety.
• Achieved high accuracy (95 %) in identifying anxiety using the RBRF algorithm on a large dataset.

## Graphical abstract

**Figure:**
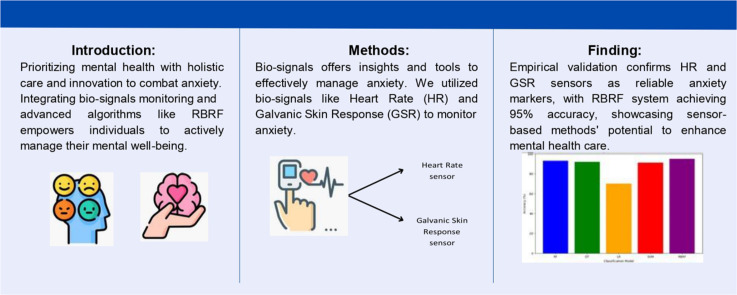

**Specifications table**

**Table:** 

**Subject area**	Computer Science
**More specific subject area**	Machine learning, IoT
**Name of your method**	RBRF (Regression Based Random Forest)
**Name and reference of original method**	Random Forest
**Resource availability**	Yes(https://github.com/Guitarcoder-dp/Bio-signal-Induced-Emotion-Monitoring-and-Detection-of-Anxiety)

## Background

Since mental health has a significant impact on both individuals and society as a whole, it is becoming increasingly recognized as a crucial aspect of overall well-being [1]. According to World Health Organization (WHO) estimates, one in four people globally may at some point in their lives develop neurological or mental illnesses [2]. The prevalence of mental health issues such as anxiety and depression highlights the importance of a comprehensive approach to mental health. The application of biosensors has changed the way we think about and approach the monitoring of mental health [3].

This work offers analytical solutions as well as a novel method for tracking emotions utilizing GSR and HR sensors. GSR sensors measure changes in skin conductance and offer data on emotional reactions in general and anxiety-related contexts. Heart rate is constantly recorded by HR sensors, providing a dynamic image of emotional states. When combined with sensors, the ESP32 microcontroller board’s versatility enables biosignal-based emotion tracking. The way that GSR and HR fluctuate during anxiety is of special interest to researchers because it sheds light on the psychological changes that occur under stressful conditions [4]. Volunteers wear the sensors while engaging in everyday tasks, allowing data collection on their physiological reactions to stress and relaxation-inducing situations. This guarantees the system will operate on schedule.

This study aims to capture the more subtle aspects of anxiety by using signal processing techniques that make use of these bio-signals [5]. The goal of the paper is to develop a strong framework for accurate anxiety state recognition and real-time emotional monitoring by concentrating on the dynamic changes in HR and GSR associated with anxiety. This method has a lot of potential for proactive intervention tactics and customized anxiety management in wearable technology and healthcare areas.

A thorough investigation of stable emotion recognition algorithms is presented in this work. Section I discusses the importance of stable algorithms as well as the reasons behind the study. A comprehensive review of the literature is done in Section II to set the study in the context of earlier studies. In Section III, the issue of instability in emotion recognition algorithms is discussed, along with its applicability and goals for future research. The methodology, which includes the research design, data sources, algorithmic frameworks, and experimental protocols, is covered in detail in Section IV. In Section V, the practical implementation is explained, along with the list of steps and challenges. In Section VI, the experiment results are analyzed and visually presented, and their implications are discussed within the context of the problem statement. In conclusion, Section VII provides a thorough summary of the main findings.

## Method details

Mental health conditions like stress, anxiety, and depression present significant obstacles for both individuals and society. Better results and an improved quality of life can come from early identification and treatment of certain illnesses. Recent advancements in technology and data analytics offer opportunities to leverage physiological signals for the early detection and monitoring of levels of anxiety. The objective of this paper is to create a predictive model that classifies people’s anxiety levels based on physiological data, specifically HR and GSR. In order to uncover unique characteristics linked to anxiety, the model will examine patterns in HR and GSR data.This will allow for the accurate and real-time classification of people’s mental states.

### Anxiety detection through electroencephalogram (EEG) signals

Anxiety detection via EEG involves analyzing brain electrical activity patterns to discern anxiety states. By leveraging EEG signals, researchers aim to identify neural markers associated with anxiety, potentially enabling personalized interventions [6]. Ural et al. [7] explored Ensemble machine learning models using modified CNN and VGG16 classifiers to accurately detect and compare major psychiatric mood disorders using non-invasive EEG signals, aiding clinicians and researchers in managing mental health. Athavipach et al. [8] developed a cost-effective, single-channel, dry contact EEG for healthcare applications, accurately categorizing basic emotions and offering the potential for home-based or telemonitoring systems, with potential for wireless connectivity. Ngai et al. [9] proposed a multi-modal approach that uses 2-channel EEG signals and the eye modality in addition to the face modality to enhance the recognition performance using convolution neural networks on bio-signal data, utilizing four batches of convolution layers and max pooling layers for each data modality.

### Anxiety detection through electrocardiogram (ECG) signals

Analyzing Electrocardiogram (ECG) signals can identify anxiety by looking at cardiac parameters and heart rate variability. With the use of this non-invasive technique, anxiety-related physiological reactions can be better understood, allowing for early detection and customized treatments [10]. Betti et al. [11] developed a wearable sensor system, using ECG, EDA, and EEG, that successfully captured human stress during the Maastricht Acute Stress Test. Achieving 86 % accuracy with an SVM classification algorithm, the study demonstrated correlations between physiological changes and salivary cortisol levels, suggesting applications for portable systems to prevent stress-related consequences. Mukhopadhyay et al. [12] used smartphones and portable data acquisition devices, a cloud-based ECG transmission system allows for real-time monitoring and visualization. This type of healthcare solution is dependable and reasonably priced, and it is especially helpful in emergency and rural situations.

### Anxiety detection through HR

A possible indicator of anxiety states is variations in heart rate dynamics, which are monitored in the process of detecting anxiety by heart rate analysis [13]. In the study conducted by Siirtola et al. [14], emotion recognition utilizing physiological signals such as HR and skin conductance is explored. This methodology offers valuable insights into customers’ emotional states, thereby facilitating marketing research and the identification of Ekman’s six primary emotions. Rakshit et al. [15] presents a method for recognizing human emotions using HRV features and SVM based classification, achieving an average recognition accuracy of 83 %. Mukhopadhyay et al. [16] provided a telemedicine solution that uses Firebase for data transfer and combines smartphones and pulse oximeters to transmit patient vitals in realtime while in transit. Their analysis assesses network performance between different cellular generations, highlighting the usefulness of 4 G for delivering healthcare remotely. Mukhopadhyay et al. [17] described a telemedicine system that helps with remote healthcare by using wireless technology to send vital signals to an Android app. For quick pre-hospital care, it places a strong emphasis on real-time heart rate monitoring from emergency vehicles to urban areas.

### Anxiety detection through skin conductance

Electrodermal Activity sensors, also known as GSR sensors, can measure continuous skin conductance changes, which are associated with sweat gland activity. These sensors offer a direct means of capturing physiological responses linked to emotional arousal, facilitating real-time monitoring of anxiety levels for personalized interventions [18]. Zamkah et al. [19] enhancing the sensitivity of future affective sensors by examining skinconductance biomarkers during stressful experiences, focusing on anti-stress hormones and cortisol metabolites as stress biomarkers. Kritikos et al. [18] emphasizes the importance of allowing patients to interact with stimuli in VR treatments for Anxiety Disorders using an EDA Sensor, enhancing treatment effectiveness and ensuring accurate biosensor measurements. Apostolidis et al. [20] proposed a study involving integrating students’ affective feedback, such as frustration, boredom, and anxiety, into learning tools and emotional appraisal learning models. By analyzing physiological reactions like skin conductivity, HR, and respiration rate, several researchers [[21], [22], [23], [24], [25], [26], [27], [28]] have assesed anxiety disorder of patients.

## Data acquisition

Our dataset was collected from 40 voluntary participants, including friends, classmates, and family members aged 12 to 57 years. The inclusion criteria required participants to be healthy and willing to engage in monitored activities, while individuals with known cardiovascular or skin-related conditions were excluded. Data collection sessions were conducted in home and classroom environments, where participants performed various daily activities such as sleeping, watching television, surfing the internet, exercising, working on a computer, and using a mobile phone. Each session lasted approximately 30 to 60 min.

To ensure proper identification and maintain data integrity, a unique User_ID was assigned to each participant, serving as the primary key in the dataset. For participants with multiple recording sessions, a Session_ID was generated by combining the User_ID with a session number (e.g., U001_S001, U001_S002).

During each session, GSR (Galvanic Skin Response) and HR (Heart Rate) signals were continuously recorded using sensors interfaced with an ESP32 microcontroller at a sampling rate of 1 Hz (one sample per second). The continuous data streams were segmented into 10-second windows with a 50 % overlap, resulting in a total of 23,915 samples across all participants. Each segment was labeled based on the participant’s activity and behavioral context under five categories: “Good,” “Least Risky,” “Less Potentially Risky,” “Moderately Risky,” and “Highly Risky.” The collected data were securely stored in Google Sheets for subsequent preprocessing and model development.

The Table 1 shows the notations that have been used in the equations.

**Table 1:** Notation table of the equation.

Symbol	Definition
ε	Error Coefficient
ω(n)	Output Signal
$Corr_{HR-GSR}$	Correlation coefficient
$HR_{t}$	Heart rate at a given time
α	Anxiety Predictive model
σ(x)	Sigmoid function
I	Anxiety Level
y	Polynomial Regression
AQ	Anxiety Quotient

The Eq. (1) quantifies the error in a predictive model by calculating the difference between the actual values u(n) and the predicted values v(n). For each index n, the squared difference between the expected and actual values is measured by the term (v(n)−u(n))2 and is summed over the whole range.

(1) $$\varepsilon =\sum_{n=1}^{N}{\left(\upsilon \left(n\right)-u\left(n\right)\right)}^{2}$$

The process of integrating sensor data (µ(n)) with a filter (η(j)) to produce an output signal ω(n) is shown in below Eq. (2). Here, the output signal at index η is denoted by ω(n), which is the product of the sensor data(µ(n − j)) and the filter coefficients (η(j)).

(2) $$\omega \left(n=\sum_{j=1}^{N}\eta \left(j\right)\mu \left(n-j\right)\right)$$

The Eq. (3) calculates Karl Pearson’s correlation coefficient [29] between HR and GSR values. Where HRi and GSRi represent the HR and GSR of the ith individual, µHR and µGSR are the means of HR and GSR across all samples, and N represents the total number of samples. The numerator computes the covariance between HR and GSR, while the denominator calculates the product of their standard deviations. This yields the Pearson correlation coefficient, which measures the strength and direction of the linear relationship between HR and GSR.

(3) $$Corr_{HR-GSR}=\frac{\sum_{i=1}^{N}\left(HR_{i}-\mu_{HR}\right)\left(GSR_{i}-\mu_{GSR}\right)}{\sqrt{{\sum_{i=1}^{N}{\left(HR_{i}-\mu_{HR}\right)}^{2}\left(GSR_{i}-\mu_{GSR}\right)}^{2}}}$$

The Eq. (4) represents the autoregressive component of the Autoregressive Integrated Moving Average (ARIMA) model. It describes how the heart rate (HR) at a given time (t) depends on its past values up to a certain lag (p), represented by autoregressive coefficients (ϕ1, ϕ2, . . ., ϕp). The term ϵt denotes the error or residual at time t. Essentially, this equation models the linear relationship between the current heart rate and its past values, allowing for forecasting and analysis of time-dependent data.

(4) $$HR_{t}=\phi_{1}\times HR_{t-1}+\phi_{2}\times HR_{t-2}+...+\phi_{p}\times HR_{t-p}+\in_{t}$$

The anxiety predictive model is indicated by Eq. (5). It uses a sigmoid function to squash output to a range between 0 and 1, with a weight vector W and a bias term b representing changes in decision boundaries. The formula determines the expected likelihood of anxiety for a given input, allowing classification using the acquired parameters W and b.

(5) α=σ(W·v+b)

The below Eq. (6) represents the sigmoid function.

(6) $$\sigma \left(x\right)=\frac{1}{1+e^{-x}}$$

In the below Eq. (7) the value of I is found by adding 1 to the total of HR and GSR, and the result is rounded to the closest integer using the Round function. In the context of a measurement or analysis where discrete values are required, this equation may be used to calculate a value based on certain physiological inputs.

(7) I=[HR+GSR]

The Eq. (8) represents a Polynomial Regression model with Interaction Terms. In this context, x represents the class label, while y represents all other features. The model captures complex relationships between the class label and other features, incorporating polynomial terms up to degree n to model non-linear relationships. Additionally, the term $\beta_{\text{int}}xy$ introduces interaction effects, allowing the model to account for changes in the relationship between the class label and features based on their joint values.

(8) $$y=\beta_{0}+\beta_{1}x+\beta_{2}x^{2}+...+\beta_{n}x^{n}+\beta_{\text{int}}$$

The relationship between anxiety (I) and the sensor readings of $HR(S_{HR})$ and GSR $\left(S_{GSR}\right)$ is represented by the Eq. (9). These sensor readings are inputs into the RBRF algorithm, which outputs the anxiety level. This formula illustrates how our method uses sensor data to precisely identify anxiety

(9) $$I=R_{BRF}\left(S_{HR},S_{GSR}\right)$$

## Algorithm

It’s essential to understand the importance of anxiety prediction in the psychology and healthcare domains before moving on with the algorithm. Early detection holds paramount importance as it facilitates the implementation of more effective intervention strategies. The following algorithm describes a method for predicting anxiety levels using a dataset D, a set of classifiers labelled as X, as well as specific training and test sets, P and Q, respectively (Algorithm 1).

**Algorithm 1:** Algorithm for predicting anxiety.

1. Input: Dataset *D* = {d1, d2, d3, . . ., dN}, set of classifiers *X* = {x1, x2, x3, . . ., xN}, training set P, test set Q
2. Let *N* = n(D)
3. Let *i* = 1
4. R(i) is a random subset of P, R(i) ⊆ P
5. M(0) = {}
6. For *j* = 1 to N:
(a) If *j* > 1:
i. r(j) = set of incorrectly classified instances of M(j−1)+R(j)
ii. M(j) = Model trained using X(j) and R(j)
7. For *j* = 1 to N:
(a) S(j) = Q classified by C(j)
8. Output: Result = max(S(j); *j* = 1, 2, . . ., N)

The first step of the procedure involves initializing variables and setting for a dataset D, a collection of classifiers X, a training set P, and a test set Q. After that, a random subset R(i) is chosen from the training set P. It then performs model training and evaluation for each classifier $x_{j}$ by training a model M(j) with a combination of the misclassified examples from the previous model and the current random subset R(j). The trained models M(j) are then evaluated on the test set Q, and the results are stored in S(j). The algorithm outputs the result at the end, choosing the maximum among the outputs from each classifier.

## Proposed architecture

Our methodology is designed to address the pressing need for accurate and efficient detection of anxiety levels using physiological data obtained from HR and GSR sensors. The well-being and quality of life of individuals are affected by anxiety disorders, which pose a serious worldwide health concern. Subjective self-reporting is a common component of traditional anxiety evaluation techniques; however, it can be biased and inaccurate. On the other hand, our method uses objective physiological markers to give a quantitative assessment of anxiety levels, providing information about how people’s bodies react to emotional and exhausting situations.

1. GSR and HR data: The first phase of our investigation entails meticulously gathering physiological data with GSR and HR sensors. An HR sensor measures the regular action of the heart, whereas a GSR sensor records variations in skin conductance, which are often associated with emotional arousal. The integration of these two data streams Fig. 1: Architecture Diagram forms the basis of our anxiety detection method, which provides a comprehensive understanding of the individual’s physiological responses to various stimuli.

**Fig. 1:**
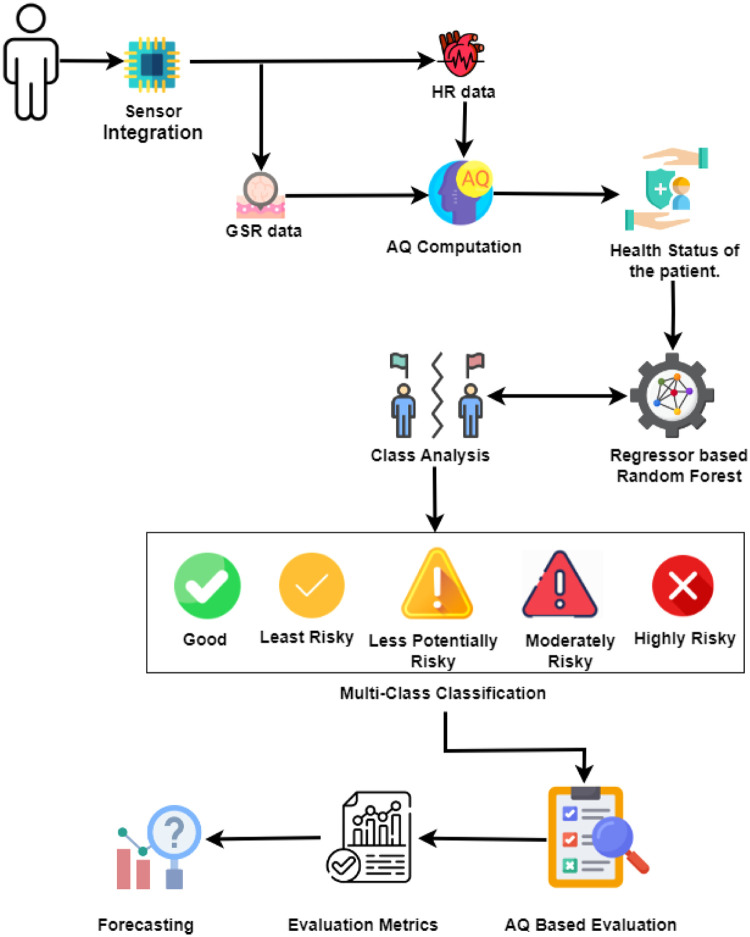
Architecture diagram.
2. Anxiety Quotient (AQ) Computation: Our study is centered on the calculation of the AQ, a measure that emerges from the interaction between HR and GSR. The average GSR is represented by GSR¯ and the average HR is represented by HR¯ . The Anxiety Quotient is calculated using equation 10. This involves scaling the result by 100 after dividing the average HR by the average GSR.

In order to make understanding easier, this method adjusts the HR to GSR ratio by a factor of 100. Apart from stress, there are other factors that might cause HR to rise; GSR reacts particularly to emotional shifts. By combining these parameters, the AQ formula seeks to produce a more complex evaluation of the physiological activation associated with anxiety. The likelihood of anxiety increases with higher anxiety levels. The participant’s physiological arousal level is gauged by the AQ, which forms the foundation for the subsequent anxiety classification.

3. Patient’s Health Status: Identifying the participant’s level of anxiety requires first completing an assessment of their overall health. Using AQ thresholds, we looked into classifying individuals into preliminary health status categories. These boundaries were set after a statistical analysis of the AQ distribution and its relationship to the results of clinical evaluation. The objective of this stage was to use the physiological data to produce a preliminary indication of the health state. The assessment comprises a thorough examination of the gathered GSR and HR data in order to determine baseline physiological parameters and spot any abnormalities or irregularities. Comprehending the participant’s initial health state is essential for placing the following anxiety tests in context and guaranteeing the validity of our results.
4. Regression-based Random Forest and Class Analysis: Introducing our innovative algorithm, RBRF. It revolutionizes the conventional approach by integrating an auto-regressor, akin to linear regression, to seamlessly generate preliminary results. Subsequently, these results undergo refinement through an RF model, culminating in exceptional accuracy rates. The calculated AQ scores and the contextual features served as the model’s regressors (input variables). The target variable was the individual’s class representing their clinically assessed anxiety level. Our algorithm RBRF, is specifically designed for anxiety prediction and is tailored to our dataset.
  The bidirectional arrow between the model and health status determination indicates that the model’s predictions might have been used to refine the preliminary health status categories derived from AQ thresholds and it involves examining the distribution and characteristics of anxiety levels within the dataset, providing valuable insights into the prevalence and severity of anxiety among participants.
5. Multi-class Classification: Our study aims to group participants into distinct anxiety levels according to their calculated Anxiety Quotient (AQ) scores. Depending on their level of anxiety, participants in the multi-class classification task are grouped as Good (G), Least Risky (LR), Less Potentially Risky (LPR), Moderately Risky (MR), or Highly Risky (HR). The categorization method draws boundaries between different anxiety levels using machine learning algorithms to aid in targeted therapies. This approach provides a more thorough assessment of anxiety levels than binary classification and may provide crucial insights into individual variations and subtle fluctuations in anxiety levels.
6. AQ Based Evaluation: One of the most important steps in our research process is AQ value evaluation. In this study, self-reported anxiety levels are compared with participants’ expected AQ scores. By analyzing the relationship between self-reported and expected anxiety levels, we assess the accuracy and efficacy of our anxiety detection system.
7. Evaluation Metrics: We assess the effectiveness of our multi-class classification model using a variety of assessment criteria, including confusion matrix analysis, accuracy, precision, recall, and F1-score. These assessments provide comprehensive information about how successfully the model classified the participants’ anxiety levels. We would like to perform a comprehensive analysis of our categorization model to identify its benefits and drawbacks to increase the accuracy and reliability of our forecasts.
8. Forecasting: Predicting future patterns in anxiety levels is critical to creating preemptive intervention plans and quickly reaching out to the most vulnerable. Using past HR and GSR data, forecasting looks for patterns and trends in anxiety levels over time. Time-series analytic tools like ARIMA models and exponential smoothing techniques can be used to anticipate future anxiety levels using past data. Our goal is to provide medical practitioners with important data for better management and intervention of anxiety-related illnesses by predicting patterns of anxiety.

## Implementation details

Galvanic Skin Response Sensor: Emotional states are closely associated with increases in skin electrical conductivity, and GSR sensors are well known for their ability to detect these changes. By detecting subtle physiological indicators associated with anxiety, these sensors provide important information about the body’s emotional condition.

Heart rate Sensor: Real-time monitoring of an individual’s heart rate is possible due to the HR sensor used in this study. Anxiety or heightened emotions may be indicated by changes in heart rate. Observing this important trait can help you get an understanding of how the body responds to various emotional situations.

ESP32 Microcontroller Board: The ESP32 board, which seamlessly integrates the HR and GSR sensors, is the brains behind our idea. Data may be gathered, stored, and analyzed in real time with this flexible microcontroller board. Based on its strong qualities, the paper will be able to collect and process biosignal data accurately.

The implementation procedure is extensive and contains plenty of important data. The first step’s primary focus is on sensor integration, which comprises physically attaching and setting the GSR and HR sensors to the ESP32 board. These sensors enable the real-time collection of physiological data, which forms the basis of our thorough analysis. After sensor integration, the paper enters the critical phase of data collection and storage. The bio-signal information gathered from the integrated sensors is safely recorded and kept in Google Sheets, where it forms the basis for additional investigation.

The fundamental aim of our study is to analyze recorded biosignal data using state-of-the-art algorithms. These intricate algorithms’ main objective is to provide predictive models that are suitable for identifying anxiety. Our algorithms recognize symptoms associated with anxiety in particular and deliver accurate and quick insights into people’s emotional states based on a vast amount of physiological data. We carry out a precise evaluation in order to guarantee the performance of our models. Confusion measures are used in the evaluation process to assess how well the models detect anxiety levels using bio-signal data. By taking this critical step, we can improve the accuracy and dependability of our algorithms in detecting minute changes in physiological reactions associated with anxiety.

The Random Forest (RF), Decision Tree (DT), Logistic Regression (LR), Support Vector Machine (SVM), and Random Forest (RBRF) approaches were selected after careful consideration of numerous aspects for our multiclass classification paper. The groups denoted by our risk evaluation labels (”Good”, ”Least Risky”, ”Less Potentially Risky”, ”Moderately Risky”, and ”Highly Risky”) exhibit varying levels of complexity and imbalance. These algorithms were chosen due to their demonstrated ability to handle problems involving multiple classes of classification.

Since RF builds many decision trees and integrates their outputs to reduce overfitting and improve classification accuracy, it can handle large, high dimensional datasets. Decision trees facilitate the perception and comprehension of decision-making processes, which can provide crucial insights into the factors influencing risk levels. Logistic regression is a well-established statistical technique that provides probabilistic interpretations of class membership and is effective in binary and multi-class classification applications. SVM is used because of its ability to handle non-linear decision constraints via kernel methods, which allows it to capture complex correlations in the data. Combining an auto-regressor module with an RF model, RBRF provides an improved approach for anxiety detection research. Regression techniques are integrated with ensemble learning in this novel methodology, improving accuracy and facilitating real-time data processing.

## Method validation

Our thorough research efforts resulted in a significant advancement in the field of anxiety detection. The combination of GSR and HR sensors, which are closely connected to the ESP32 board, makes it possible to collect vital physiological data in real-time. Our efforts have been remarkably successful in accurately detecting anxiety, mainly due to the use of machine learning techniques such as RF, Decision Tree, Logistic Regression, SVM, and RBRF. Notably, our RBRF, which has an astounding 95 % accuracy rate, highlights the effectiveness of our method. The dataset was divided into training and testing sets with a split ratio of 80:20.

**Fig. 2:**
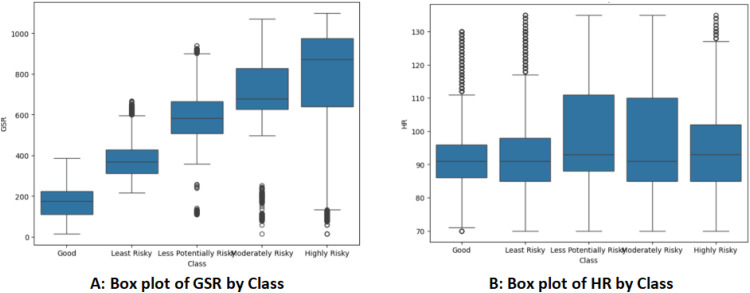
Box plot of (A) GSR and (B) HR by class.

Fig. 2 represents box plots using the Seaborn library that visualize the distribution of HR and GSR measurements across different classes within the dataset. The x-axis displays different classes present in the dataset, while the y-axis represents the GSR measurements in Fig. 2(A) and GSR measurements in Fig. 2(B).

**Fig. 3:**
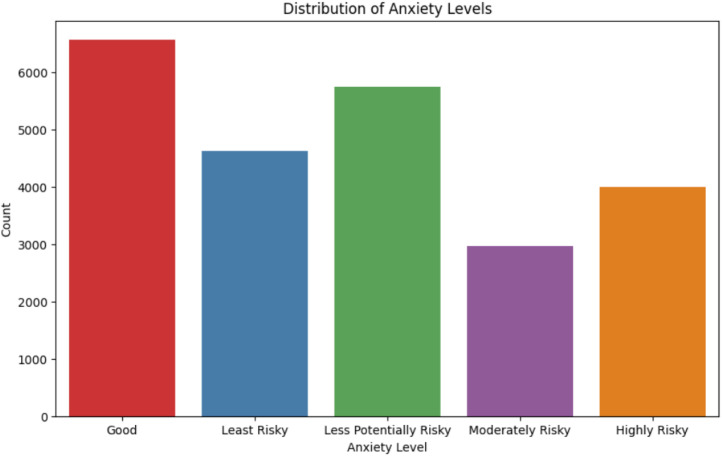
Distribution of anxiety levels.

Fig. 3 represents a count plot using the Seaborn library to visualize the distribution of anxiety levels across different classes within the dataset. Where the x-axis represents the different anxiety levels present in the dataset, and the y-axis represents the count of occurrences for each class.

**Fig. 4:**
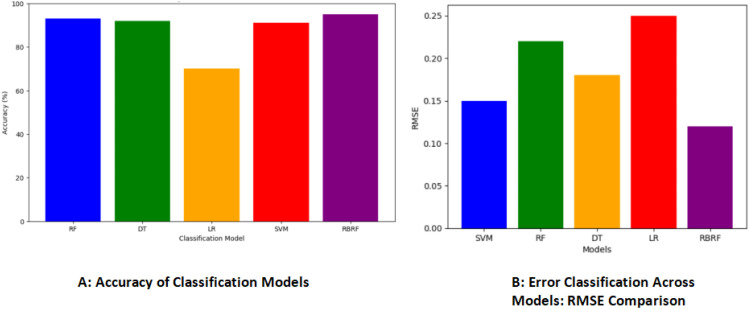
Bar graph of (A) Accuracy and (B) Error classification across models.

Fig. 4 represents a bar graph using Matplotlib to illustrate the accuracy and RMSE of different classification models. In 4(A) each bar in the graph represents the accuracy of a specific model, with different colors as signed to differentiate between models. The x-axis displays the names of the classification models, while the y-axis represents the accuracy percentage. To compare the efficacy of different machine learning models, 4(B) uses the Root Mean Square Error (RMSE). The RMSE metric is widely used to assess model accuracy because it measures the average magnitude of errors between predicted and actual values. As the RMSE decreases, the predictive accuracy of the model rises. The relative positions of models on the graph can be used to assess each model’s ability to minimize errors, which can aid in selecting the most appropriate model for a given task.

The outcomes of our models are particularly noteworthy, showcasing impressive accuracy rates. The changes are carefully examined and visualized using graphical illustrations [30]. Specifically, our RBRF model achieved an accuracy of 95 %, the RF model achieved an accuracy of 93 %, and the Decision Tree model demonstrated an accuracy of 92 %. The SVM model surpassed expectations with an accuracy rate of 91 %, and the Logistic Regression model achieved an accuracy of 70 %. These results underscore the effectiveness of our approach and signify a substantial leap forward in comprehending biosignal-based emotional states, particularly anxiety.

**Fig. 5:**
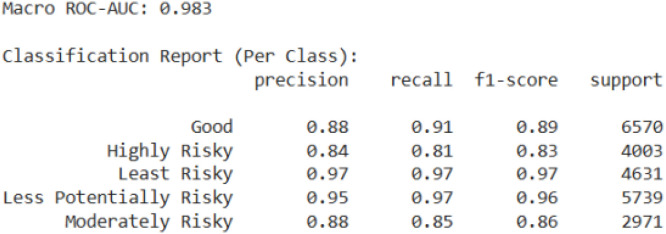
ROC-AUC curve and classification report of the RBRF.

Fig. 5 represents the ROC curve and classification report of the RBRF, demonstrating strong predictive performance with high accuracy and balanced class-wise metrics.

**Fig. 6:**
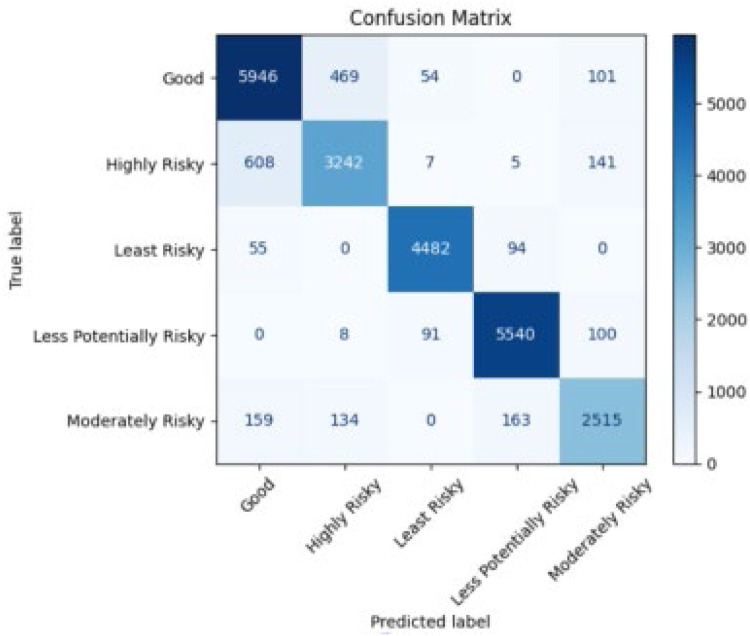
Confusion matrix representing class-wise classification results.

Fig. 6 represents the confusion matrix showing the class-wise classification results of the RBRF. It highlights how accurately each risk category—Good, Highly Risky, Least Risky, Less Potentially Risky, and Moderately Risky—was predicted, demonstrating the model’s effective performance across multiple classes.

**Fig. 7:**
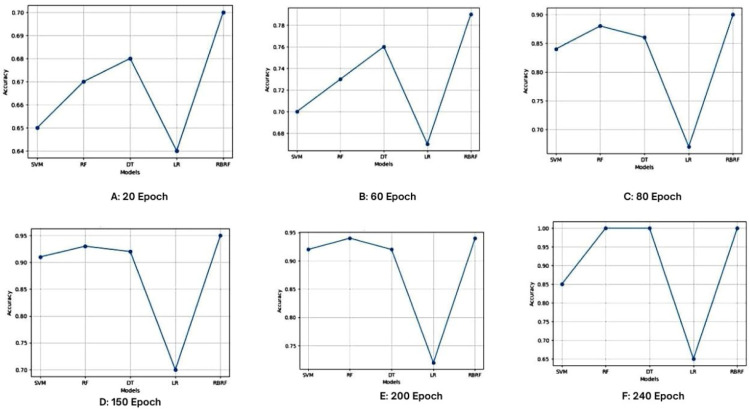
Accuracy of classification models.

The performance trends of models RF, Decision Tree, SVM, Logistic Regression, and RBRF over various epochs are depicted in Fig. 7 the gradual evolution of model accuracy over epochs is evident. The accuracy scores are plotted on the y-axis, and the models are plotted on the x-axis. Every curve relates to a particular machine learning model and offers information about how that model learns and how its performance changes over epochs.

With an increase in the number of epochs, several of the models in Fig. 7(F) exhibit overfitting. This suggests that some of the models in the plot may have a tendency to overfit the training set as the training iterations progress, which could result in the capture of noise or specific patterns that are not representative of the dataset as a whole.

## Limitations

• Limited generalizability due to individual differences such as age, gender, and medical conditions.
• Environmental factors like temperature and humidity can affect GSR and HR readings.
• Absence of psychological validation through self-reported anxiety levels or clinical diagnosis.
• Focuses on short-term physiological responses without considering long-term anxiety patterns.

## Ethics statements

Human subject appear in our research work. All participants provided informed consent prior to their involvement in the study. The study was conducted in compliance with ethical guidelines, ensuring participant confidentiality and data protection.

## CRediT author statement

**Adwitiya Mukhopadhyay**: Supervision, Methodology,Conceptualization, Writing- Original draft preparation, Software. **Divyashree D P**: Writing- Reviewing and Editing. **Ramya C A**: Visualization, Investigation. **Hijaz Ahmad**: Conceptualization. **Taha Radwan**: Software, Validation. **Soumik Das**: Validity tests, Data curation, Writing- Original draft preparation.

## Consent form

I ____________________________ give my consent to collect the data and use it for the project named "Bio Signal-Based Emotion Monitoring and Detection of Anxiety." I understand the purpose of the data collection and how my data will be used. I understand that my participation is voluntary. I understand that my data will be kept confidential and used only for the stated purposes.

Participant's Signature:

## Declaration of competing interest

The authors declare that they have no known competing financial interests or personal relationships that could have appeared to influence the work reported in this paper.

## Data Availability

Data can be accessed from the source file
